# Rapid detection of *sacbrood virus *(SBV) by one-step reverse transcription loop-mediated isothermal amplification assay

**DOI:** 10.1186/1743-422X-9-47

**Published:** 2012-02-17

**Authors:** Yang Jin-Long, Yang Rui, Shen Ke-Fei, Peng Xiang-Wei, Xiong Tao, Liu Zuo-Hua

**Affiliations:** 1 Chongqing Academy of Animal Science, Chongqing 402460, China; 2 Rongchang Bureau of Animal Husbandry, Chongqing 402460, China

**Keywords:** *Sacbrood virus*, Loop-mediated isothermal amplification, SYBR Green, Honeybee

## Abstract

**Background:**

*Sacbrood virus *(SBV) primarily infects honeybee broods, and in order to deal with the problem cost effective detection methods are required.

**Findings:**

A one-step reverse transcription loop-mediated isothermal amplification (RT-LAMP) assay was developed for the rapid identification of SBV. The data demonstrated that, in a simple water bath, SBV RNA could be detected as early as 20 min at 65°C, and a positive amplification reaction was visible to the naked eye due to a color change brought on by the addition of nucleic acid stain SYBR Green.

**Conclusions:**

The current study presents a method for the rapid and simple detection of SBV by RT-LAMP with high sensitivity and analytic specificity.

## 

*Sacbrood virus *(SBV) primarily affects honeybee broods, and results in larval death. Infected larvae change color from pearly white to pale yellow, and shortly after death they dry out, forming a dark brown gondola-shaped scale [1]. Suitable detection methods are needed to control and eradicate SBV. Several have been developed, such as immunodiffusion assays, radioimmunoassay, enzyme-linked immunosorbent assay (ELISA), and qualitative PCR [2]. However, in contrast to these assays, reverse transcription loop-mediated isothermal amplification (RT-LAMP) does not require expensive or special equipment [3,4]. Therefore, LAMP-based detection assays would be suitable for on-the-spot detection in the field or primitive laboratories. The aim of this study was to develop a novel method for the detection of SBV in a simple, rapid and cost-effective manner.

A set of six specific primers was designed by targeting the sequence of the SBV-pol gene sequence (GenBank accession no. AF092924.1) using Primer Explorer version 3 (http://primerexplorer.jp/e/intro/index.html). The nucleotide sequences of the primers are shown in Table 1. A brood of honeybees known to be infected with *Sacbrood virus *(SBV) were kindly provided by Dr. S. KF from Chongqing Academy of Animal Science. All samples were stored at -20°C before RNA extraction. Total RNA was extracted from SBV isolates using an RNA extraction kit (TaKaRa Biotechnology, Dalian, China) according to the manufacturer's protocol. Total RNA was resuspended in water and quantified by spectrophotometry [5]. The RT-LAMP reaction was carried out in a 25 μl reaction mixture containing 2 μM each of the inner primers FIP and BIP, 0.2 μM each of the outer primers F3 and B3, 1.4 mM deoxyribonucleotide triphosphate (dNTP) mix (TaKaRa Biotechnology, Dalian, China.), 5 mM MgSO_4_, 16 units of *Bacillus stearothermophilus *(*Bst*) DNA polymerase (New England Biolabs Inc., Ipswich, MA), 1 × the supplied *Bst *DNA polymerase buffer, 0.125 units of AMV reverse transcriptase, and 2 μl of template RNA. The RT-LAMP reaction mixtures were incubated at the optimal reaction temperature (65°C) for the optimal reaction time (50 min) and were finally heat inactivated at 85°C for 2 min to terminate the reaction.

**Table 1:** The reverse transcription loop-mediated isothermal amplification (RT-LAMP) primer sets

Primer	Type	5'pos	3'pos	Length	Sequence(5' to -3')
F3	Forward outer	519	539	21-nt	AAGGAACTATAGTATGGCGAA

B3	Backward outer	707	724	18-nt	CTGTTGCTGGTCTCTTGT

FIP	Forward inner	592	616	46-mer(F1c:25-nt,	TGGACCTACAAATTGCACCAATATA-
	(F1c + F2)	548	568	F2:21-nt)	ACCTCTTACAGTTGCAAAGTG

BIP	Backward	619	640	44-mer (B1c:22-nt,	AAGGACCCAGAGTGATGAGGTA-
	inner(B1c + B2)	679	700	B2:22-nt)	TGTATTTTCTTCCTTGGAACTT

LF	Loop Forward	569	591	23-nt	CTCTTAGCTGCTAGTTCTGAAGC

LB	Loop Backward	641	663	23-nt	CCCTCGAAAGAATCTATTCAGGG

The RT-LAMP assay successfully amplified the target sequence of the SBV-pol gene, as observed by 2% agarose gel electrophoresis. The effect could also be seen by the naked eye on addition of 1.0 μl 1,000-fold diluted original SYBR Green I (Molecular Probes, Inc.). The solution changed from light orange to green in the presence of LAMP amplicons, while it remained light orange in the absence of amplification. Amplified DNA in the LAMP reaction causes white turbidity due to the accumulation of magnesium pyrophosphate, a by-product of the reaction. Prior to the addition of SYBR Green I, the white turbidity of the reaction mixture by magnesium pyrophosphate was also inspected [3].

Temperature and reaction duration are critical parameters in RT-LAMP reaction. To determine the optimal reaction temperature, the RT-LAMP reactions were carried out for 60 min at 59°C, 60°C, 61°C, 62°C, 63°C, 64°C and 65°C. The DNA products from all reactions except that done at 59°C showed an obvious ladder-like pattern on the gel. The intensities of the DNA products at 63°C, 64°C and 65°C were higher than those at other temperatures (Figure 1). Therefore, 63°C-65°C was considered the optimal temperature range for RT-LAMP reaction for the detection of SBV.

**Figure 1:**
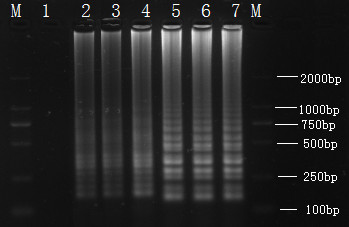
**Determination of the optimal temperature of the LAMP**. Determination of the optimal temperature. **Lane M**, DL-2000 DNA marker; **Lanes **1-7: LAMP carried out at 59, 60, 61, 62, 63, 64 and 65°C.

To determine the optimal reaction time, the total reaction mixture was incubated at 65°C for 10, 20, 30, 40, and 50 min. The DNA products from the reaction with a duration of between 30 and 50 min showed the highest intensity and the earliest detection time was 20 min (Figure 2). Therefore, 30-50 min was considered the optimal reaction time range for the RT-LAMP reaction. The final, optimal RT-LAMP protocol was therefore determined to be for a time of between 30 and 50 min at a temperature in the range 63°C-65°C.

**Figure 2:**
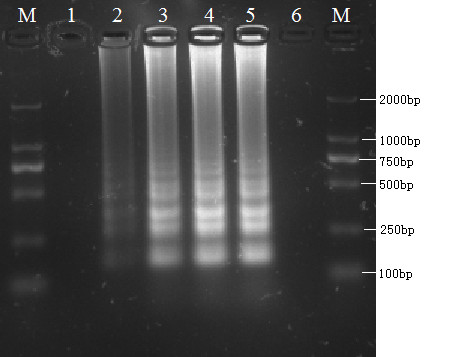
**Determination of the optimal time of the LAMP**. **Lane M**, DL-2000 DNA marker; **Lanes 1-5: **LAMP carried out for 10, 20, 30, 40 and 50 min, respectively; **Lane 6: **-, negative control. All products were electrophoresed on 2% agarose gels and stained with ethidium bromide.

The RT-LAMP product was detected using the naked eye by observing the white turbidity of the reaction mixture (Figure 3A) or color change of the solution when stained with SYBR Green I (Figure 3B).

**Figure 3:**
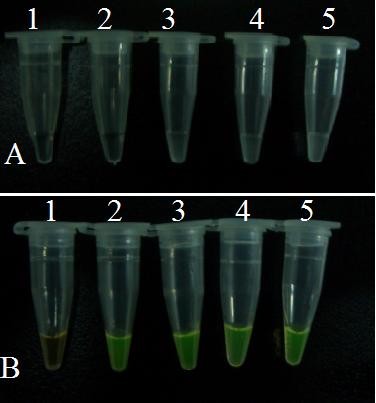
**Detection of LAMP products by observing white turbidity and color of the reaction mixture**. **(A) **Shows white turbidity of the reaction mixture by magnesium pyrophosphate; **(B) **Shows color (green) of the reaction mixture after adding SYBR Green I. **1-5**, reaction carried out using 10-fold serial dilutions of standard SBV DNA (1.0 × 10^4 ^copies/μL): 1: 1.0 × 10^0^, 2: 1.0 × 10^1^, 3: 1.0 × 10^2^, 4: 1.0 × 10^3^, and 5: 1.0 × 10^4 ^copies/μL, respectively.

Figure 3A shows that white turbidity was observed in reaction products when using samples with between 1.0 × 10^2 ^copies/μl to 1.0 × 10^4 ^copies/μl of standard template RNA. However, this was not observed in samples with between 1.0 × 10^0 ^to 1.0 × 10^1 ^copies/μl.

In Figure 3B, after adding 1 μl of diluted SYBR Green I to the reaction tube, the color of the RT-LAMP reaction solution changed from orange to green in samples with between 1.0 × 10^1 ^copies/μl to 1.0 × 10^4 ^copies/μl of standard template RNA. No color change was observed in 1.0 × 10^0 ^copies/μl.

Taken together, these results show that the RT-LAMP detection limit is 1.0 × 10^2 ^copies for white turbidity assay, and 1.0 × 10^1 ^copies for analysis with SYBR Green I. Therefore, we conclude that color observation method (using SYBR Green I) was ten times more sensitive than the white turbidity observation.

The specificity of the RT-LAMP assay was determined with SBV isolates and other honeybee viruses (deformed wing virus (DWV), chronic bee paralysis virus (CBPV), Kashmir bee virus (KBV)). All SBV strains were positive while all other honeybee viruses were negative. This demonstrates that the RT-LAMP assay is specific, with no cross-reaction with other honeybee viruses.

To evaluate the application of RT-LAMP to detect SBV in clinical samples, the test was performed on 30 field clinical samples collected in Chongqing, China, in the period March to May 2011. These were obtained from broods in apiaries with unusually high mortality, which was suspected to be due to SBV infection. The tests yielded 27 positive and three negative results by both RT-LAMP and RT-PCR assays [6].

In summary, the current study presents a method for the rapid detection of SBV by RT-LAMP with high sensitivity and analytic specificity. The assay is feasible for use in less well-equipped laboratories as well as in the field.

## Competing interests

The authors declare that they have no competing interests.

## Authors' contributions

JY, YR and KS carried out most of the experiments and wrote the manuscript, and should be considered as first authors. XP and ZL critically revised the manuscript and the experiment design. TX helped with the experiment. All of the authors read and approved the final version of the manuscript.
